# Operative management of cryptorchidism: guidelines and reality - a 10-year observational analysis of 3587 cases

**DOI:** 10.1186/s12887-015-0429-1

**Published:** 2015-09-10

**Authors:** Kai O. Hensel, Tawa Caspers, Andreas C. Jenke, Ekkehard Schuler, Stefan Wirth

**Affiliations:** Department of Pediatrics, HELIOS Medical Center Wuppertal, Children’s Hospital, Centre for Clinical & Translational Research (CCTR), Faculty of Health, Witten/Herdecke University, Heusnerstr. 40, D-42283 Wuppertal, Germany; Institute for Quality Management, HELIOS Kliniken GmbH, Berlin, Germany

**Keywords:** Undescended testis, Testicular descent, Primary cryptorchidism, Maldescensus testis, Retractile testis, Acquired cryptorchidism, Orchidopexy, Timing of surgery, Health services research, Guideline implementation

## Abstract

**Background:**

Undescended testis (UDT) is the most common disorder in pediatric surgery and one of the most important risk factors for malignancy and subfertility. In 2009 local guidelines were modified and now recommend treatment to be completed by the age of 1. Aim of this study was to analyze age distribution at the time of orchidopexy, whether the procedure is performed according to guideline recommendations and to assess primary care pediatricians’ attitude regarding their treatment approach.

**Methods:**

We retrospectively analyzed 3587 patients with UDT regarding age at orchidopexy between 2003 and 2012 in 13 German hospitals. Furthermore, we conducted an anonymized nation-wide survey among primary care pediatricians regarding their attitude toward management of UDT.

**Results:**

Before modification of the guideline 78 % (*n* = 1245) of the boys with UDT were not operated according to guideline recommendations. After the modification that number rose to 95 % (*n* = 1472). 42 % of the orchidopexies were performed on patients aged 4 to 17 years. 46 % of the primary care pediatricians were not aware of this discrepancy and 38 % would only initiate operative management after the first year of life. In hospitals with pediatric surgery departments significantly more patients received orchidopexy in their first year of life (*p* < .001).

**Conclusion:**

The guideline for UDT in Germany has not yet been implemented sufficiently. Timing of orchidopexy must be optimized in order to improve long-term prognosis. Both primary care providers and parents should be educated regarding the advantages of early orchidopexy in UDT. Prospective studies are needed to elucidate the high rate of late orchidopexies.

**Electronic supplementary material:**

The online version of this article (doi:10.1186/s12887-015-0429-1) contains supplementary material, which is available to authorized users.

## Background

Primary undescended testis is the most common congenital anomaly of the urogenital system and the most common disorder in pediatric surgery, affecting up to 30 % of preterm and 3 % of term infants worldwide [1–3]. A synopsis of nomenclature and possible origins of non-scrotal position of the testis is presented in Table 1. The non-scrotal position of the testis bears a considerable risk for the development of both uni- and contra-lateral testicular malignancy as well as impaired fertility [4, 5]. Testicular decent takes place at two stages under control of insulin-like hormone 3 between 8 and 15 weeks of development [6] and androgens facilitating inguinoscrotal migration afterwards [7]. While plenty of research is dedicated to improve understanding of the morphological complexity involved in the process of testicular descent, the exact cause of cryptorchidism currently remains elusive. Relevant risk factors include prematurity, genetic predisposition, endocrine disorders (e.g. disrupted hypothalamic-pituitary-gonad axis), small-for-gestational-weight (SGA), birth weight < 2500 g as well as environmental factors (nicotine, alcohol, pesticides) [2, 8–11]. 10 % of the cases are bilateral and are commonly associated with complex syndromes or other congenital malformations such as abdominal wall defects or neural tube defects [9, 12]. Spontaneous descent occurs in approximately 70 % of the cases, mostly within the first three (to six) months of life [13–15]. After six months of life therapeutic intervention is indicated, as a spontaneous descent is then unlikely. Hormonal treatments with GnRH monotherapy or in combination with ß-HCG yield success rates of 15–20 % [16–18]. However, secondary re-ascent occurs in approximately 20 % of the successfully treated boys and a positive effect of hormonal therapy on the degree of paternity remains to be scientifically proven [19]. Scrotal or inguinal orchidopexy is the surgical treatment of choice in prepubertal boys with palpable, cryptorchid testes [20, 21]. In case of nonpalpable testes an examination under anaesthesia by a surgical specialist is indicated. If the testis remains unpalbable, surgical exploration and laparoscopic abdominal orchidopexy are the treatment of choice [22]. Surgery bears a high success rate and complications occur infrequently (1–3 %) [16, 23]. In UDT, early orchidopexy has been proven to improve prognosis regarding testicular growth, number of germ cells and the risk for malignant transformation [24, 25]. Recently, experts even suggested surgery to take place as early as within the first three to nine months of life in order to prevent abnormal gonocyte maturation in the affected testis [7]. Neoadjuvant GnRH treatment has been shown to improve the fertility index in prepubertal UDT and is thus thought to improve fertility later in life [26]. A prospective randomized trial published by Spinelli et al. in 2014 reported that patients with UDT and a testicular atrophy index > 20 % had a significant increase in testicular volume after 5 years of follow-up when treated with pre- and postoperative GnRHa therapy [27]. Consequently, treatment recommendations for UDT can be assumed to remain dynamically changing in the near future.

**Table 1:** Synopsis of nomenclature and etiology for non-scrotal testes

Medical term	Meaning	Possible origin
Cryptorchidism	“Hidden testis”, extra- /supra-scrotal position	• Agenesis, atrophy [2]
		• No/delayed testicular descent
Undescended testis	Incomplete descent of the testis, possible positions: intra-abdominal, inside the inguinal canal or supra-scrotal	• Immaturity, low birth weight [2, 8]
Retractile testis	Normal testicular position, periodic translocation to a supra-scrotal position	• Hyperactive cremasteric reflex [47, 48]
Ascending testis, acquired undescended testis	Previously regular positioned testis, secondary permanent translocation to a non-scrotal position	• Deviating growth velocity of spermatic chord and body length growth [50]
		• Partial absorption of the vaginal process into the peritoneum [50]

In Germany, the first official treatment guideline for UDT (AWMF-register, no. 006/022) from 1999 targeted orchidopexy to be performed within the first two years of life. In 2009 this guideline recommendation was modified, indicating that operative treatment has to be completed by the end of the first year of life [28]. This modification was preceded by consensus statements of several international expert consortiums in 2008 [29, 30]. The importance of early orchidopexy in undescended testis can be expressed by the fact that age at orchidopexy has been suggested as a general indicator of the quality of regional child health services [31].

Aim of this retrospective study was to analyze age distribution at orchidopexy and whether timing of operative treatment in patients with UDT in Germany is managed according to guideline recommendations. Furthermore, we investigated whether the guideline modification concerning earlier timing of orchidopexy has been implemented in day-to-day clinical routine. In addition, a nationwide survey was carried out to assess the primary care pediatricians’ attitude regarding operative management of UDT.

## Methods

### Patients

All orchidopexies (*n* = 5462) performed in all HELIOS hospitals in Germany between 2003 and 2012 were assessed. All pediatric cases of uni- or bilateral UDT (*n* = 3587) were analyzed with regard to age distribution for the entire period of time as well as for the individual years from 2003 to 2012. Indications other than UDT (e.g. testicular torsion) (*n* = 1486) and hospitals with < 100 orchidopexies per year (17 hospitals, *n* = 389) were excluded from the analysis. In order to allow time for the implementation of the modified treatment recommendation, the year 2009 was excluded from the comparison analysis of the two guideline validity periods. The study was carried out in compliance with the Helsinki Declaration and ethical/medical data protection approval was obtained from the Helios Research Medical Controlling Council. Given the retrospective design of this study, the need for consent was waived.

### Nationwide survey of primary care pediatricians

Supported by the German Professional Association of Pediatricians (BVKJ), we conducted a nationwide, anonymized, web-based online survey of primary care pediatricians comprising all 16 federal states of Germany from June to December 2013. Primary care providers were randomly selected from a database provided by the BVKJ including - at the given time - all practicing primary care pediatricians in the country. 127 (response rate of 16 %) of the 811 invited pediatricians participated. The survey contained specific questions concerning therapeutic management of pediatric patients with UDT. Particular emphasis was placed on timing of operative intervention. Contact details were obtained through the public website http://www.kinderaerzte-im-netz.de/aerzte/suche.html. The questions asked are presented in Fig. 3 (Additional file 1).

### Statistical analysis

Primary endpoint of this retrospective cross-sectional study was a documented orchidopexy of patients with UDT in their first year of life according to current guideline recommendations. The confirmatory analysis was based on a two-sided Fisher test (5 % level of significance) comparing the relative frequencies of children operated in accordance to versus contrary to guideline recommendations, respectively; in addition, an approximate 95 % confidence interval for the difference of these frequencies was estimated. Furthermore, in terms of exploratory evaluations, a stratification of this comparison for hospitals with and without a department of pediatric surgery was conducted. Respective Fisher tests and confidence intervals were then performed at the local 5 % significance level without correction for multiplicity. The anonymized survey was analyzed descriptively; its results were reported with absolute and relative frequencies. All analyses were conducted with SPSS® (Version 21.0 for Windows®). For significance and confidence validation the software R® was utilized.

## Results

### Timing of orchidopexy in patients with undescended testis

**Fig. 1:**
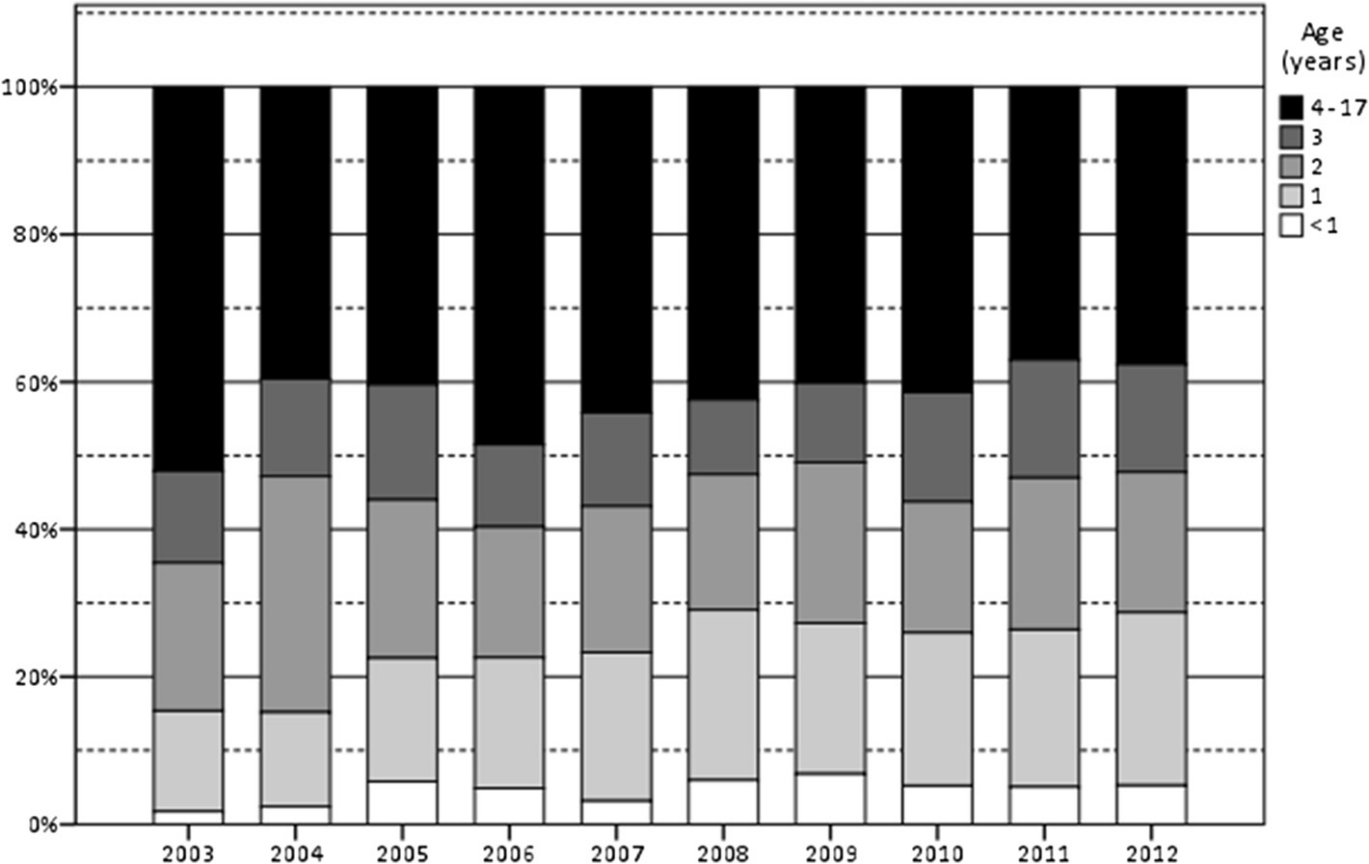
Relative age distribution at the time of orchidopexy for the years 2003 to 2012

**Fig. 2:**
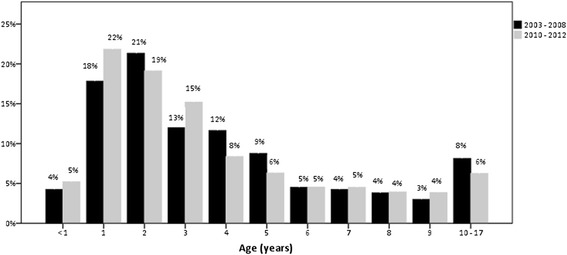
Distribution of age at orchidopexy prior to versus following the guideline modification (black: 2003–2008, grey: 2010–2012; *n* = 3587)

Table 2 and Fig. 1 show the age distribution of all boys that received orchidopexy because of UDT between 2003 and 2012 (*n* = 3587). 41 % of the patients were older than 4 years. From 2003 to 2008 4 % were operated before age 1 and 22 % before the age of 2. After the guideline recommendation was modified (including one year tolerance) only 5 % were operated in their first year of life and 27 % before the age of 2*.* Respective 95-% confidence intervals were [−3 %; +1 %; corresponding Fisher *p* = .209] for the incidence difference of children operated in their first year of life and [−13 %; −5 %; corresponding Fisher *p* < .001] for the first two years of life. Figure 2 demonstrates the age distribution at the time of orchidopexy of all cases from 1 to 17 years of age before and after change of the guideline recommendation, respectively. Taking into consideration only boys aged 4 years and younger, only 8 % received orchidopexy in the first year of life from 2003 to 2008 and 9 % from 2010 to 2012.

**Table 2:** Age distribution of patients with undescended testis at the time of orchidopexy before and after modification of the guideline recommendation (due to statistical rounding not all percentages add up to 100 %)

Age (years)	0	1	2	3	4	5	6	7	8	9	10–17
2003 – 2012	*n* = 179	713	733	482	351	272	174	148	141	136	258
	5 %	20 %	20 %	13 %	10 %	8 %	5 %	4 %	4 %	4 %	7 %
2003 – 2008	*n* = 68	285	341	199	186	140	72	68	61	48	130
	4 %	18 %	21 %	13 %	12 %	9 %	5 %	4 %	4 %	3 %	8 %
2010 – 2012	*n* = 81	339	297	236	130	98	84	70	61	60	97
	5 %	22 %	19 %	15 %	8 %	6 %	5 %	5 %	4 %	4 %	6 %

While an average of 266 total cases have been operated each year between 2003 and 2008 (*n* = 1598) in all analyzed hospitals, that number rose to 518 cases per year between 2010 and 2012 (*n* = 1553). There has been a decrease in the general population number in Germany from 82,5 million inhabitants in 2003 to 80,5 million in 2012 [32] and an increase in the incidence of orchidopexies in the analyzed hospitals from 3,2/1.000.000 to 6,4/1.000.000 (*data not shown*).

The influence of the presence of a pediatric surgery department on the timing of orchidopexy in UDT is presented in Table 3. Without exception, more patients were operated according to guideline recommendations in hospitals with a department of pediatric surgery both before and after modification of the guideline (Fisher *p* < .001).

**Table 3:** Cases of orchidopexy stratified according to hospitals with and without a department for pediatric surgery prior to (2003–2008) and after (2010–2012) modification of the guideline, respectively; *p*-values indicate the difference of cases < 1 year (≤2 years) of age and all other cases. CI = 95-% confidence interval

		Cases	Hospitals with a department for pediatric surgery	Hospitals without a department for pediatric surgery	*p*-value [CI]
		*n* = 1598	*n* = 660	*n* = 938	
2003 – 2008	<1 year	*n* = 68	*n* = 41	*n* = 27	*p* = .001 [1 %;6 %]
			6 %	3 %	
	≥1 year	*n* = 1530	*n* = 619	*n* = 911	
			94 %	97 %	
	<2 years	*n* = 353	*n* = 192	*n* = 161	*p* < .001 [15 %;26 %]
			29 %	17 %	
	≥2 years	*n* = 1245	*n* = 468	*n* = 777	
			71 %	83 %	
2010 – 2012		*n* = 1553	*n* = 508	*n* = 1045	
	<1 year	*n* = 81	*n* = 50	*n* = 31	*p* < .001 [5 %;11 %]
			10 %	3 %	
	≥1 year	*n* = 1472	*n* = 458	*n* = 1014	
			90 %	97 %	
	<2 years	*n* = 420	*n* = 159	*n* = 261	*p* = .008 [6 %;18 %]
			31 %	25 %	
	≥2 years	*n* = 1133	*n* = 349	*n* = 784	
			69 %	75 %	

### Nationwide survey of primary care pediatricians

**Fig. 3:**
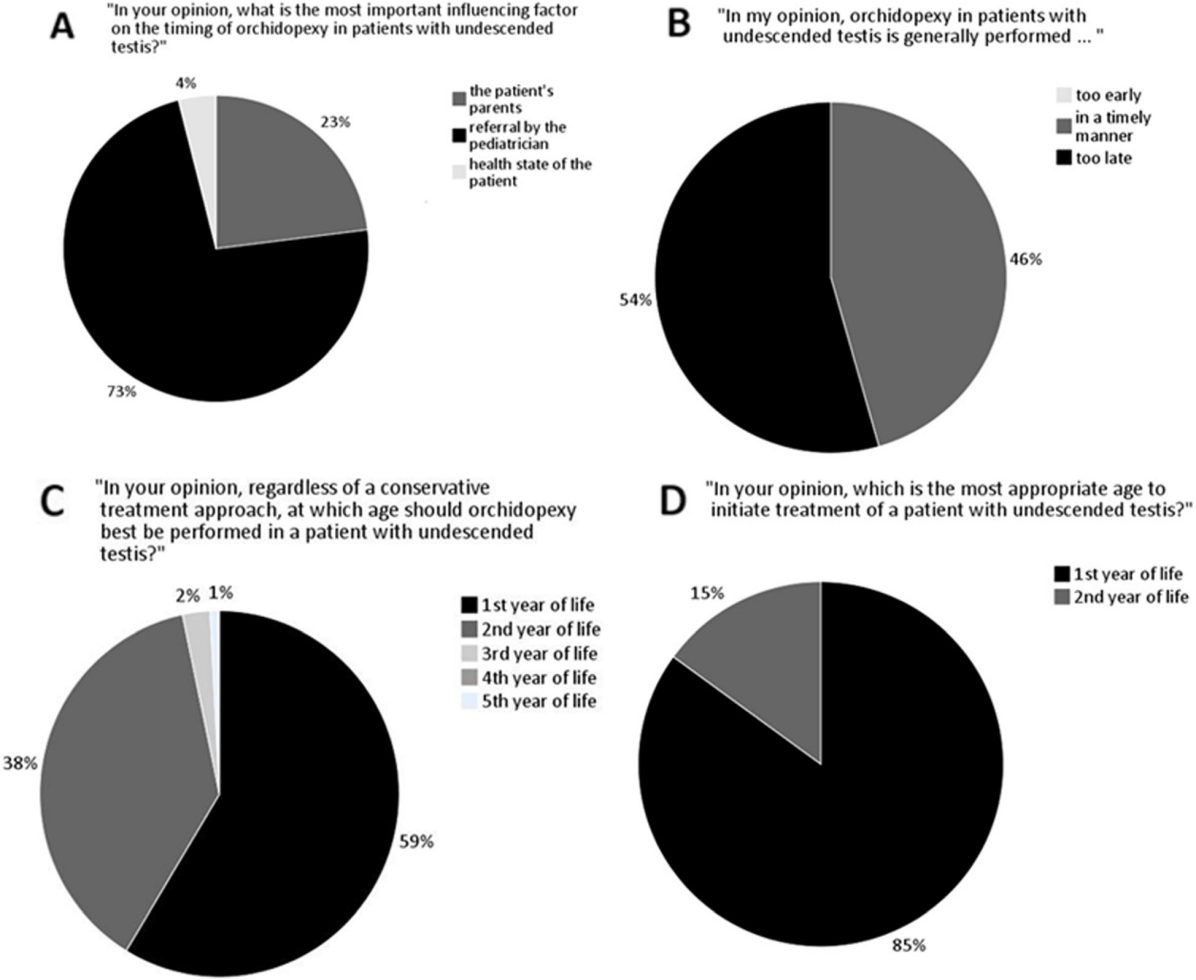
**a** Survey response results to the question concerning the most important influencing factor regarding the timing of orchidopexy of patients with undescended testes (*n* = 126). **b** Survey response results to the question, whether surgical management of orchidopexy is generally performed in a timely manner (*n* = 125). **c** Survey response results to the question regarding the appropriate age to perform orchidopexy on a patient with undescended testis (*n* = 127). **d** Survey response results to the question concerning the ideal time point to first initiate treatment in a boy with undescended testis (*n* = 127)

73 % of the responding pediatricians consider the referral of the treating primary care pediatrician the most important influencing factor for the timing of operative management in UDT. 23 % mentioned the parents’ decision as most influential (Fig. 3a). 54 % of the respondents documented the average timing of orchidopexy in UDT to be delayed while 46 % were in the opinion that on average surgical treatment is performed in a timely manner (Fig. 3b). 59 % considered the first year of life as the optimal period for orchidopexy in UDT, 38 % refer for surgery in the second year of life (Fig. 3c). 15 % of the primary care pediatricians would only initiate treatment - regardless whether conservative or surgical - after the first year of life (Fig. 3d). The question which treatment modality should primarily be initiated in patients with UDT was answered with “conservative treatment” by 82 % and “surgical procedure” by 17 % of the pediatricians (*data not shown*).

## Discussion

The aim of this study was to investigate whether surgical repair of UDT in Germany is performed according to medical guideline recommendations and whether the modified recommendation (advocating treatment is to be completed within the first year of life) has been implemented in clinical day-to-day routine. Furthermore, we conducted a survey elucidating primary care pediatricians’ attitude toward operative management of UDT.

We included a total of 3587 cases of UDT over a 10-year period in 13 HELIOS hospitals distributed throughout Germany. To date, no comparable data set has been published. Our results show that from 2003 to 2012 the average timing of orchidopexy in UDT deviates clearly from the guideline recommendation. From 2003 to 2008, 78 % of the patients were not operated according to the medical guideline, namely, after their second year of life. After modification of the guideline recommendation 95 % of the orchidopexies were performed after the first year of life. This is in line with findings from a German university hospital, published 2012 by Höfling et al. as well as with data from an Austrian study from 2010 [33, 34]. Similarly, in the USA, only 18 % of the patients with UDT received orchidopexy before the age of 2 and 43 % were operated before reaching the age of 3 years between 1999 and 2008, as one study reported [35]. An Australian study about orchidopexy in UDT in the state of Victoria from 1999 to 2006 demonstrated that while the overall orchidopexy rate declined by 26 %, the percentage of boys aged 0 to 2 years rose from 44 % to 58 % [36].

These data point out an important shortcoming in pediatric health care, as it is well known that a delay in definite management of UDT leads to increased rates of subfertility, malignant transformation and testicular torsion [37, 38].

Despite the marked overall delay in operative treatment of UDT on the one hand, our data, on the other hand, reveals also a positive trend regarding the timing of surgery. The guideline modification in 2009 lead to a small but statistically significant increase in orchidopexies before the age of 3 (27 % versus 22 %). Even though this is an important step in the right direction, the current state still essentially bears improvement potential [39].

We observed an increase in the incidence of orchidopexies in the included hospitals of this study from 3,2/1.000.000 per year (2003–2008) to 6,4/1.000.000. This can only partly be explained by the continuous decrease of the general population number from 82,5 million in 2003 to 80,5 million inhabitants in 2012. Clearly, treatment numbers of the analyzed hospitals are increasing as hospitals are expanding which leads to rising numbers of treated. However, since we have analyzed 13 hospitals of a specific private hospital chain the increase in incidence of orchidopexies should not be mistaken for a general increase rate for this operation. In addition, the rise in early orchidopexies after changing of the guidelines logically dictates, that cases that would have been operated later in life and thus not in the analyzed period have in fact been operated earlier, hence resulting in an increase in orchidopexies immediately following the change of guidelines. This effect will probably slightly fade in the following years. However, since the relative amount of cases operated in a timely manner according to guidelines shows a positive tendency, the absolute numbers are not as significant as the percentage of the cases treated within the first year of life.

Strikingly, our results show a statistically significant influence of the presence of a department for pediatric surgery on the timing of orchidopexy in UDT. In hospitals with pediatric surgery departments, significantly more children received surgical treatment according to guideline recommendations; both before (29 % vs. 17 %) as well as after modification of the guideline (10 % vs. 3 %). Even though it is no secret that mutual reservations concerning medical interventions in young patients are somewhat ubiquitous, it yet seems surprising that the above mentioned impact is that distinct. However, even though statistically significant, the number of patients undergoing orchidopexy before the age of 1 is still extremely low.

There are several valid explanatory approaches for the discrepancy specified above. Provided that all cases of UDT are actually primary in nature, possible influencing factors are screening failure, a lack of knowledge among referring health care providers, timing problems with referral to subspecialties, the unwillingness of parents to have a surgical procedure performed on their child at a young age, the surgeons’ endeavor to perform surgery in patients of an older age in order to avoid complications associated with immaturity as well as the general health state of the patient himself.

To analyze treatment strategies of primary care providers, we carried out a nationwide survey. Our results show that delayed referral by primary care pediatricians may in fact be an important influencing factor accounting for the high rate of late orchidopexies of patients with UDT. While 54 % think that operative treatment generally takes place too late, 46 % of the respondents consider the average timing of orchidopexy to be in accordance with guideline recommendations. 38 % refer for surgical treatment of UDT no earlier than in the second year of life - in other words, 38 % would only initiate surgical treatment after it is suggested to be already completed according to the medical guideline. These results are in line with findings from a study from 2010, in which 82 pediatricians in the south of Germany were interviewed with regard to their attitude toward surgical management of UDT [33]. Furthermore, 15 % of the responding pediatricians in our study would not initiate any form of medical or surgical treatment within the first year of life. 73 % consider referral for surgery by primary care providers to be the most important factor influencing timing of orchidopexy in UDT. This emphasizes the impact of the primary care pediatricians’ attitude toward surgical management and thus guideline implementation in UDT. However, the response rate of 16 % in this survey is a limitation to the generalization of these findings.

Contrary to our expectations, 1480 (>41 %) of the here documented patients were ≥ 5 years old when they received orchidopexy for UDT. Is a wide-ranging screening failure the reason for this high rate of late orchidopexies? It has only gradually been accepted over the last two decades that beside the primary (congenital) form of UDT, a non-scrotal position of the testis can also be acquired (ascending testes, secondary cryptorchidism). A study published in 2003 in the Netherlands postulated acquired UDT to occur three times as often as the more commonly known primary form [40]. Until the 1980s secondary ascent of the testis was largely unknown and publications dealing with this topic were titled “Ascent of the Testis: Fact or Fiction” and consisted merely of individual case reports [41, 42]. Motivated by the unexplained advanced age at orchidopexy (mean age: Lamah et al.: 5,5 years; Hack et al.: 6,6 years) several studies have covered the “enigma” of the high rate of late orchidopexies in UDT [40, 43]. In a study from 2013 by van der Plas et al. 660 cases of non-scrotal testis managed with orchidopexy after the age of 2 were assessed [44]. In more than two thirds of the cases (*n* = 421), the previous position of the testes was documented several times before in the clinical records. 34 % of these cases were primary UDT. For 278 boys (66 %), however, a previous scrotal position of the non-scrotal testes had been documented at least twice before (secondary ascent of the testis). Given the retrospective nature of this study, the significance of these findings may be somewhat limited by inter-observer variation (inconsistency in physical examination and record documentation) and selection bias, as a large number of patients was excluded due to lack of information regarding their previous medical records.

Guven and others have investigated possible reasons for the high rate of late orchidopexies in a study from 2008 [45]. 33 % of the analyzed patients received orchidopexy after the age of 4 years. 46 % had the diagnosis of acquired UDT. In 22 % of the cases the parents were the reason for the late operation and 9 % were iatrogenic. In 85 % of the cases of acquired UDT the testes were previously documented to be retractile. This is in line with findings from Agarwal et al., who demonstrated that 32 % of the cases of retractile testes will eventually lead to a secondary ascent of the testis [46]. Hyperactivity of the cremasteric reflex is currently discussed to be the most important stimulus for the non-scrotal position in retractile testes [47, 48]. A possible explanation may be the fact that the cremasteric reflex peaks in reactivity between age 5 and 8 [49]. Furthermore, the partial absorption of the processus vaginalis into the parietal peritoneum and a disproportion of growth rate of the inguinal canal in comparison to the development of body length growth [50]. Since there is a significant risk of testicular atrophy and subsequently impaired fertility even in retractile testis, it has been suggested to utilize the decrease in testicular volume – measured by ultrasonography – as a device to target surgical treatment in ambiguous cases [51].

In a review article Sijstermans et al. have analyzed 46 studies covering UDT [52]. Only 6 publications (11 %) could distinctly discriminate between primary and secondary cryptorchidism. These studies consistently demonstrate that the rate of acquired UDT is much more common than previously assumed. An Australian study claims, that presumed acquired UDT accounts for approximately 50 % of all performed orchidopexies [36]. It is therefore arguable that the here described high rate of late orchidopexies is at least partly represented by secondary ascent of the testis. Ultimately, the exact role of inadequate transposition of the guidelines versus secondary testicular ascent remains to be elucidated by further, prospective studies.

## Conclusion

The surgical management of patients with cryptorchidism needs to be improved. The attitude of 46 % of the responding primary care pediatricians does not reflect guideline recommendations with regard to the timing of orchidopexy in UDT. Even though an increasing trend was demonstrated, the recent share of boys that received surgical repair in accordance with guideline recommendations was only 5 %. In hospitals with a department for pediatric surgery, still small numbers but significantly more patients received orchidopexy according to guideline recommendations. In order to improve prognosis regarding malignancy and subfertility, both pediatricians, general practitioners and parents need to be continuously educated about the advantages of early orchidopexy in UDT. The surprisingly high rate of late orchidopexies justifies the conclusion that acquired cryptorchidism may essentially be much more common than previously expected. Prospective long-term studies are needed to further elucidate this underestimated disorder. Frequent examinations of testes and scrotum have to be documented throughout childhood at medical check-ups in order to shorten the interval between occurrence of acquired UDT and initiation of treatment.

## Additional file

Additional file 1:
**Nationwide survey of primary care pediatricians regarding their attitude toward management of pediatric patients with undescended testes.** (DOCX 16 kb)
