# Supplementary material for: Operative management of cryptorchidism: guidelines and reality - a 10-year observational analysis of 3587 cases
Source: BMC Pediatr. 2015 Sep 10;15:116. doi: 10.1186/s12887-015-0429-1 (PMC4566496; doi:10.1186/s12887-015-0429-1)
Supplement: Additional file 1: — Nationwide survey of primary care pediatricians regarding their attitude toward management of pediatric patients with undescended testes. (DOCX 16 kb) [file 12887_2015_429_MOESM1_ESM.docx]

**Nationwide survey of primary care pediatricians regarding their attitude toward management of pediatric patients with undescended testes**

A In your opinion, what is the most important influencing factor on the timing of orchidopexy in patients with undescended testis?

- The patient’s parents
- Referral by the pediatrician
- Health state of the patient

B In my opinion, orchidopexy in patients with undescended testis is generally performed...

- ...too early
- ...in a timely manner
- ...too late

C In your opinion, regardless of a conservative treatment approach, at which age should orchidopexy best be performed in a patient with undescended testis?

- 1^st^ year of life
- 2^nd^ year of life
- 3^rd^ year of life
- 4^th^ year of life
- 5^th^ year of life

D In your opinion, what is the most appropriate age to initiate treatment in a patient with undescended testis?

- 1^st^ year of life
- 2^nd^ year of life

E In your opinion, which treatment modality should primarily be initiated in patients with undescended testis?

- Conservative treatment
- Surgical procedure
